# Identification of antagonistic fungi and their antifungal activities against aconite root rot pathogens

**DOI:** 10.1080/15592324.2023.2211852

**Published:** 2023-05-15

**Authors:** Ran Liu, Maoting Chen, Jing Gao, Min Luo, Guangzhi Wang

**Affiliations:** School of Pharmacy, Chengdu University of Traditional Chinese Medicine, Chengdu, China

**Keywords:** Aconitum carmichaelii Debx, root rot disease, antagonistic fungi, antagonistic activity, Trichoderma

## Abstract

Root rot is one of the main diseases affecting Aconitum carmichaelii Debx. during cultivation, seriously limiting yields of this herb. Currently, there is no effective control measure for aconite root rot. The antifungal activities of antagonistic strains against aconite root rot pathogens (Fusarium proliferatum, Fusarium solani, and Fusarium oxysporum) were investigated in this study. Three antagonistic strains, JKT7, JKT28 and JKT39, were screened and identified as Trichoderma asperellum, Trichoderma hamatum and Trichoderma virens, respectively. Dual culture tests showed that the inhibition rates of the three Trichoderma strains on the pathogens were all approximately 70%. The volatile metabolites had inhibitory effects on the mycelial growth of pathogens, while the nonvolatile metabolites in the culture filtrates did not show significant inhibitory effects. The volatile components analyzed by GC‒MS were mainly ketones, esters, and alcohols. These results indicate that these strains of Trichoderma and their secondary metabolites have antimicrobial activities against the pathogens of aconite root rot. This study could provide a scientific basis for the biocontrol of aconite root rot.

## 1. Introduction

*Aconitum carmichaelii* Debx., a traditional medicinal plant with a long history of cultivation in Sichuan, China, belongs to the genus *Aconitum* L. in the family Ranunculaceae. Its root system is a well-known medicinal material used in China, India and other Asian countries. There are many diseases affecting *A. carmichaelii* Debx. during cultivation, including root rot, white rot (sclerotia), leaf spot, downy mildew, etc. Among them, root rot most significantly limits the yield of *A. carmichaelii* Debx.^1^ Chen et al.^2^ found that *Fusarium proliferatum*, *F. solani*, and *F. oxysporum* are the main pathogens causing root rot of *A. carmichaelii* Debx. Currently, the control of root rot mainly utilizes chemically synthesized pesticides.^3^ However, the excessive use of pesticides can easily cause environmental pollution and loss of soil fertility.^4,5^ Thus, the quality and safety of these medicinal materials are affected.^6^ In addition, pesticide residues can destroy beneficial microorganisms in the soil^7^ and increase the resistance of pathogens. An increasing number of studies have shown that biological control of plant diseases has more advantages than chemical control and is more efficient and environmentally friendly.^8–11^

As a group of important biological control fungi, *Trichoderma* have the characteristics of rapid growth^7^, diverse biological control mechanisms and rich metabolites. Studies have shown that various *Trichoderma* spp. fungi have significant effects on the control of plant diseases. The *T. harzianum* strains TvDPs, TDPs and T1s, isolated from the soil of *Phoenix dactylifera* by Perveen and Bokhari^12^, reduce the occurrence of root rot caused by *F. oxysporum*. The biological control mechanisms of *Trichoderma* include competition with the pathogen for nutrients and living space, production of metabolites with antifungal activities, and induction of resistance in plants, etc.^13–16^ Many secondary metabolites with important biological functions have been found in different species of *Trichoderma*^17^, including different classes of antifungal compounds: (*a*) volatile antibiotics, e.g., 6-pentyl-α-pyrone (6PP) and most of the isocyanide derivates; (*b*) water-soluble compounds, e.g., heptelidic acid or koningic acid; and (*c*) peptaibiotics and peptaibols.^18^ Their interactions with plants have been studied in depth, and some secondary metabolites have been found to participate in the regulation of plant growth and activate plant defense responses.^19–21^ Several studies have shown that *Trichoderma* can induce plants to produce terpenoids, phenols and other compounds in the process of interacting with host plant roots to improve plant resistance to diseases.^22–26^

In this study, *Trichoderma* strains that have strong antagonistic effects on pathogens of aconite root rot were screened from the rhizosphere soil of healthy *A. carmichaelii* Debx. plants. The biocontrol potential of these strains against aconite root rot was determined through dual culture tests, assays of the inhibition of pathogenic mycelial growth and spore germination by secondary metabolites, and GC‒MS of volatile metabolites.

## 2. Materials and methods

### 2.1. Pathogenic fungal strains and spore suspension

Three *Fusarium* strains were isolated from *A. carmichaelii* Debx. plants with root rot obtained from the GAP base in Puzhao Village, Jiangyou, China. The strains were identified as *F. proliferatum*, *F. solani*, and *F. oxysporum* based on their morphological characteristics and 18S *rDNA* gene sequencing, and they were stored on potato dextrose agar slant culture medium at 4°C.

The pathogens were grown on potato dextrose agar (PDA) medium in Petri dishes at 28°C for 3 days. Spores of pathogens were washed thoroughly with 20 mL of sterile water. The spore suspension (1.0 × 10^6^ spores/mL) of the pathogen was prepared after being shaken slightly on a shaker for 30 min and filtered with sterile filter paper to remove mycelia.

### 2.2. Isolation and purification of antagonistic strains

The soil sample was collected from the rhizosphere soil of healthy *A. carmichaelii* Debx. plants obtained from Butuo County, Yi Nationality Autonomous Prefecture of Liangshan (Sichuan, China).

The serial dilution spread plate technique^27^ was used to separate the fungi from the soil. The soil was weighed (2.5 g) into a 50 mL Erlenmeyer flask with 25 mL of sterile water. The mother liquor was obtained after shaking for 30 min at 120 rpm and standing for 30 min. One milliliter of mother liquor was taken and diluted sequentially into six gradients from 10^−1^ to 10^−6^. From each dilution, 100 μL of the solution was taken and spread evenly on a PDA plate. Sterile water was applied as a blank control. The plates were incubated at 28°C for 3–5 days. Fungal colonies were picked and streaked onto PDA plates.^27^ Pure subcultures were obtained from growing colonies on PDA and stored at 4°C until use.

A 4-mm mycelial disk of pathogen was cut from the edges of actively growing colonies and placed in the center of a PDA plate. Four different strains to be tested were inoculated at 2 cm from the mycelial disk of the pathogen. Each strain was set up with three replicates and cultured at 28°C for 5 days. Antagonistic strains were screened according to the size of the zone of inhibition.

### 2.3. Molecular identification of antagonistic strains

The genomic DNA of the antagonistic strains was extracted using the E.Z.N.A. Fungal DNA Mini Kit (Omega Bio-tek Inc., America). DNA integrity was analyzed by electrophoresis on 1% agarose gels. The primers for ITS sequencing of the fungi were ITS1 (5′-TCCGTAGGTGAACCTGCGG-3′) and ITS4 (5′-TCCTCCGCTTATTGATATGC-3′). Polymerase chain reaction (PCR) was performed in a 25-μL reaction mixture solution containing 1 μL of DNA, 1 μL each of the downstream and upstream primers, 9.5 μL of double-distilled water, and 12.5 μL 2×*Taq* PCR Master Mix (Sangon Biotech Co., Ltd., Shanghai, China). The amplification program comprised initial denaturation at 95°C for 3 min, followed by 30 cycles of denaturing at 95°C for 30 s, annealing at 54°C for 45 s, extension at 72°C for 1 min by 30 cycles, and a final extension for 10 min at 72°C. The amplified fragments were purified and sequenced by an external service provider (Sangon Biotech Co., Ltd., Shanghai, China). The sequences were used to perform a BLAST search against the National Center for Biotechnology Information (NCBI) sequence database (GenBank) to compare the sequences with the most similarity to identify each fungus. A phylogenetic tree was reconstructed, and the evolutionary history was inferred using the neighbor-joining method.^28^ Tree topologies were evaluated by performing bootstrap analysis of 1,000 datasets^29^ with MEGA 7.0.^30^

### 2.4. Inhibitory activities of antagonistic fungi against pathogens

The dual culture method^31^ was used to determine the inhibitory activities of the antagonistic strains against the root rot pathogens. The three pathogens mentioned above and the selected antagonistic fungi were activated. A 4-mm mycelial disk was cut from the edge of each actively growing colony. One mycelial disk of pathogen was placed on one side of a PDA plate, and another mycelial disk of antagonistic fungus was placed on the opposite side of the same PDA plate. The distance between the two mycelial disks was 5 cm. PDA plates inoculated with each pathogen only were used as controls. Inoculated plates were incubated at 28°C for 7 days, and each treatment was repeated three times. The radial growth of the colony of the pathogen relative to the direction of the antagonistic fungus was observed daily, and the colony radius of the pathogen was measured.

The percentage of growth inhibition in the radial growth of the pathogen was calculated using the following formula:

Percentage of growth inhibition = (C-T)/C × 100,

where C is the colony radius of the pathogen in the control group, and T is the colony radius of the pathogen in the treatment group.

### 2.5. Impacts of volatile metabolites of antagonistic fungi on pathogen growth

According to the method of Chen et al.,^32^ a 4-mm mycelial disk of pathogen and a 4-mm mycelial disk of antagonistic fungus were cut from actively growing cultures and placed on the center of different PDA plates. The lid of the plate with the antagonistic fungus was replaced by the bottom containing PDA inoculated with the pathogen. The two plates were taped together with parafilm. Pathogen grown on medium without antagonistic fungus was used as a control. The plates were incubated at 28°C for 7 days, and each treatment was repeated three times. The radial growth of the pathogen was observed, and the colony diameter of the pathogen was measured daily.

The percent inhibition in the radial growth of volatile metabolites of antagonistic fungi on pathogens was calculated using the following formula:

Percentage of growth inhibition = (C-T)/C × 100,

where C is the colony diameter of the pathogen in the control group, and T is the colony diameter of the pathogen in the treatment group.

### 2.6. Impacts of nonvolatile metabolites on pathogen growth

The activities of nonvolatile metabolites were studied by the culture filtrate method.^33^ Two plugs (4 mm each) of each antagonistic species, cut from the actively growing margins of 3-day-old cultures, were used to inoculate 250 mL Erlenmeyer flasks containing 100 mL of sterile potato dextrose broth (PDB). The liquid cultures were incubated for 7 days at 28°C under stirring (180 rpm). The cultures were centrifuged at 10,000 rpm for 20 min and then filtered through sterilized filter paper and a 0.22 µm nylon membrane to obtain cell-free culture filtrates.

Fifty milliliters of culture filtrate of the antagonistic fungus was added to 200 mL of sterilized PDA medium cooled to 50°C and well mixed. Then, the mixture was poured evenly into Petri dishes and solidified. The control set was prepared by using sterile water mixed in the same ratio in PDA medium. A 4-mm agar block of actively growing colonies from 3-day-old cultures of pathogens was cut and inoculated at the center of the above-prepared Petri plates. The inoculated Petri plates were incubated at 28°C for 7 days, and radial growth was measured daily. The percentage inhibition in the radial growth of the colony was calculated by the same formula as described in 2.5.

### 2.7. Impacts of nonvolatile metabolites on spore germination of the pathogens

The cell-free culture filtrate of antagonistic fungus and spore suspension (1.0 × 10^6^ spores/mL) of the pathogen (see section 2.1) were mixed at a ratio of 1:4. The control set was prepared by using sterile water mixed with the same ratio in the spore suspension. An appropriate amount of petroleum jelly was applied to the four corners of a sterile coverslip. A small drop of mixed liquid was dropped on the center of the coverslip, making the droplet align with the center of the groove of a sterile concave slide. Then, the concave slide was covered and quickly turned over so that the droplet did not contact the groove.^34^ In a petri dish with two layers of sterile filter paper, 10 mL of sterile water was added to keep the filter paper moist. Two sterile toothpicks were placed on the filter paper, and the concave slide was placed on the toothpicks. Petri dishes were incubated at 28°C, and the germination of pathogen spores was observed every 2 hours for 8 hours. Each treatment was repeated three times.

### 2.8. Analysis of volatile metabolites of antagonistic fungi by GC‒MS

The cell-free culture filtrate of each antagonistic fungus was extracted three times for 30 min each time with equal volumes of ethyl acetate and n-butyl alcohol. After combining the extract liquor, the ethyl acetate phase and the n-butyl alcohol phase were obtained separately. The extract liquor of each phase was concentrated to 5 mL under reduced pressure at 35–45°C. All samples were filtered through a 0.22 µm nylon membrane prior to GC‒MS.

The analysis of volatile components in culture filtrates was carried out on a GC‒MS (FULI-Chromatec Crystal 9000). An Agilent VF-624 ms column (60 m × 0.25 mm × 1.4 μm) was used with hydrogen as the carrier gas, and the flow rate was 1 mL/min. A 1 µL sample was injected in splitless mode, and the vaporizer temperature was 220°C. The oven was programmed to start at 35°C (held for 4 min) and to ramp up to 95°C at 6°C/min (held for 3 min), and then, it was increased to 230°C at 15°C/min (held for 15 min). The ion source temperature was set at 230°C. The ionizing electron energy was 70 eV, and the scan range was 35–350 u. Compounds were identified using the National Institute of Standards and Technology database version 17.0. The results were expressed as a percentage of the volatile organic compounds by dividing the area of each peak by the total area of the chromatogram peaks.^35^

## 3. Results

### 3.1. Molecular identification of antagonistic strains

After preliminary screening, three strains (JKT7, JKT28 and JKT39) with good antagonistic effects on the three main pathogens of root rot of *A. carmichaelii* Debx. were selected.

**Figure 1.**
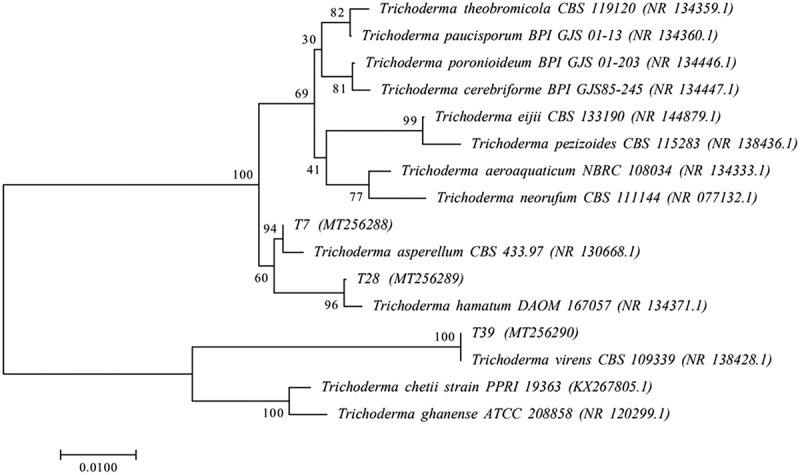
Phylogenetic tree based on rDNA-ITS sequence analysis of antagonistic fungi. The confidence values over 50% from 1,000 replicate bootstrap samplings are shown at each node. The accession numbers of strains are shown in parentheses. The scale bar indicates the base substitution rate.

The sequencing results showed that the ITS regions of the *rDNA* of samples JKT7, JKT28 and JKT39 were 599 bp, 584 bp and 621 bp, respectively, and they were submitted to the NCBI database for comparison. Their corresponding accession numbers are MT256288, MT256289 and MT256290. The homologies between JKT7 and *Trichoderma asperellum* (NR 130,668.1), JKT28 and *Trichoderma hamatum* (NR 134,371.1), and JKT39 and *Trichoderma virens* (NR 138,428.1) were all above 99%. A phylogenetic tree was constructed utilizing the neighbor-joining (NJ) method. The results are shown in Figure 1: JKT7 and *T. asperellum* (NR 130,668.1) were clustered together at 94% of the bootstrap value; JKT28 and *T. hamatum* (NR 134,371.1) were clustered together at 96% of the bootstrap value; and JKT39 and *T. virens* (NR 138,428.1) were clustered together at 100% of the bootstrap value. Therefore, JKT7, JKT28 and JKT39 were identified as *T. asperellum*, *T. hamatum* and *T. virens*, respectively.

### 3.2. Inhibitory effects of antagonistic fungi on pathogens

**Figure 2.**
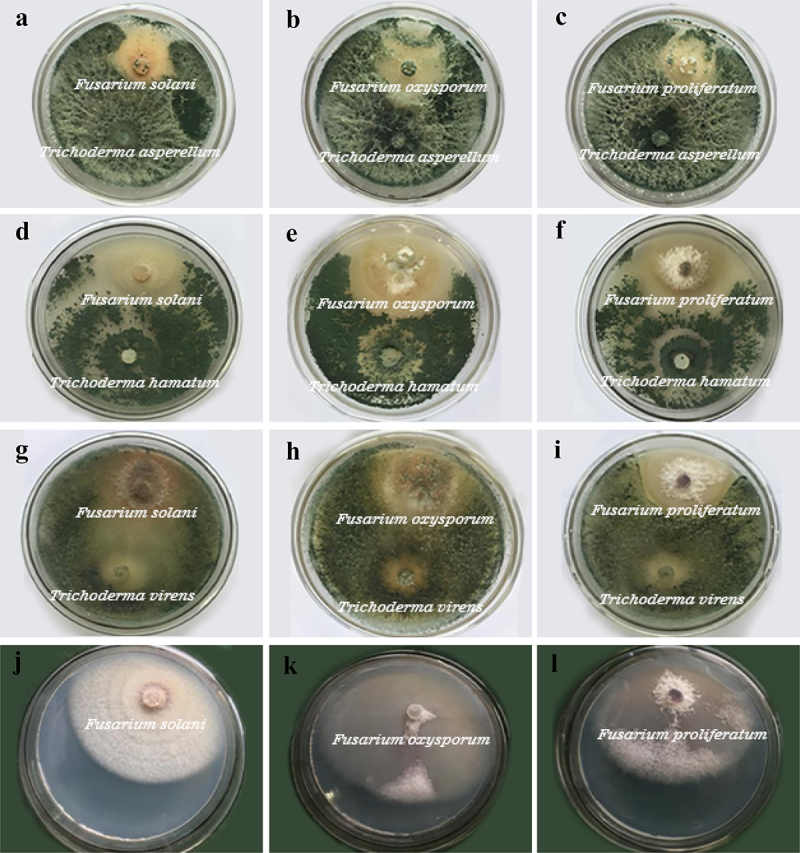
Inhibitory effects of antagonistic fungi on pathogens on the 7th day of culture. a-c, in turn, are Trichoderma asperellum against F. solani, F. oxysporum and F. proliferatum. d-f are T. hamatum against F. solani, F. oxysporum and F. proliferatum. g-i, in turn, are T. virens against F. solani, F. oxysporum and F. proliferatum. j-l are the blank controls of F. solani, F. oxysporum and F. proliferatum, respectively.

The results from the dual culture assay showed that the three strains of *Trichoderma* have significant inhibitory effects on root rot pathogens of *A. carmichaelii* Debx., and their inhibition rates were all approximately 70%, as shown in Table 1. Among them, *T. asperellum* showed obvious inhibition of the hyphal growth of pathogens, while the inhibition by *T. hamatum* was the weakest. In the dual culture assay, the growth rate of *Trichoderma* was significantly faster than that of the pathogens. On the second day of inoculation, contact between the hyphae of the two fungi could be observed, and the inhibitory effect began to appear. During incubation, *Trichoderma* gradually occupied the living space and limited the growth of the pathogen. After 4 days of cultivation, the fast-growing mycelia of *Trichoderma* grew directly on the surface of the pathogen colony, making it difficult for pathogenic mycelia to grow until they gradually atrophied, and finally, the mycelia of *Trichoderma* filled the plate. Due to inhibition by *Trichoderma*, the radial growth of the pathogen in the treatment group was significantly different from that in the control group. As shown in Figure 2, after 7 days of culture, the pathogens could not grow radially, and some pathogen colonies gradually withered.

**Table 1.** Inhibition of the growth of aconite root rot pathogens by three antagonistic fungi.

Strains	Percentage of growth inhibition		
	*F. solani* (Fus.)	*F. oxysporum* (Fuo.)	*F. proliferatum* (Fup.)
*T. asperellum*	75.51 ± 0.47a	78.23 ± 2.55a	78.73 ± 1.13a
*T. hamatum*	72.67 ± 1.24b	73.44 ± 0.72b	68.32 ± 1.85b
*T. virens*	76.65 ± 0.96a	77.31 ± 1.43a	69.80 ± 0.83b

Data in the table are mean ± standard deviation (*n* = 3). Different treatments marked with the same letters indicate no significant difference (*P* > 0.05), while different letters indicate significant or extremely significant differences (*P* < 0.05).

### 3.3. Impacts of volatile metabolites of antagonistic fungi on the growth of pathogens

**Figure 3.**
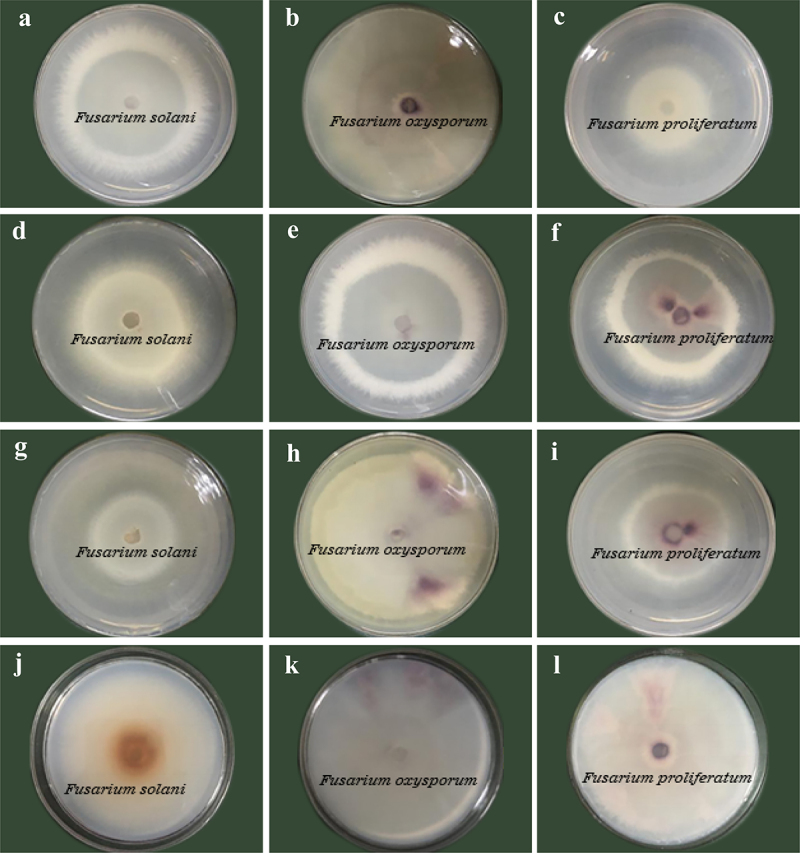
Antagonistic activities of volatile metabolites of antagonistic fungi against pathogens. a-c, in turn, are T. asperellum against F. solani, F. oxysporum and F. proliferatum. d-f are T. hamatum against F. solani, F. oxysporum and F. proliferatum. g-i, in turn, are T. virens against F. solani, F. oxysporum and F. proliferatum. j-l are the comparisons of F. solani, F. oxysporum and F. proliferatum in order.

As shown in Figure 3, the colony diameter of the pathogen in the treatment group was significantly smaller than that in the control group. The inhibitory effects started to show after 24 hours of incubation and were enhanced with increasing incubation time. The inhibitory effects were generally observed on the 3rd day of culture. On the 4th day, the inhibitory effects were significantly enhanced. As shown in Table 2, a significant inhibitory effect of *T. virens* on *F. solani* was shown on the 7th day of culture, with a rate of 62.09%. The antagonistic fungus that had a significant inhibitory effect on *F. oxysporum* was *T. asperellum*, with an inhibition rate of 37.00%. Compared with *T. hamatum*, *T. asperellum* and *T. virens* showed better inhibitory activities against *F. proliferatum* (41.73% and 42.43%, respectively).

**Table 2.** Inhibition of the growth of aconite root rot pathogens by the volatile components of antagonistic fungi.

Strains	Percentage of growth inhibition		
	*F. solani* (Fus.)	*F. oxysporum* (Fuo.)	*F. proliferatum* (Fup.)
*T. asperellum*	16.68 ± 2.14c	37.00 ± 2.99a	41.73 ± 1.25a
*T. hamatum*	47.08 ± 1.04b	12.73 ± 1.54b	25.00 ± 3.09b
*T. virens*	62.09 ± 2.37a	16.12 ± 1.62b	42.43 ± 0.97a

Data in the table are mean ± standard deviation (*n* = 3). Different treatments marked with the same letters indicate no significant difference (*P* > 0.05), while different letters indicate significant or extremely significant differences (*P* < 0.05).

### 3.4. Impacts of nonvolatile metabolites on the growth of pathogens

**Figure 4.**
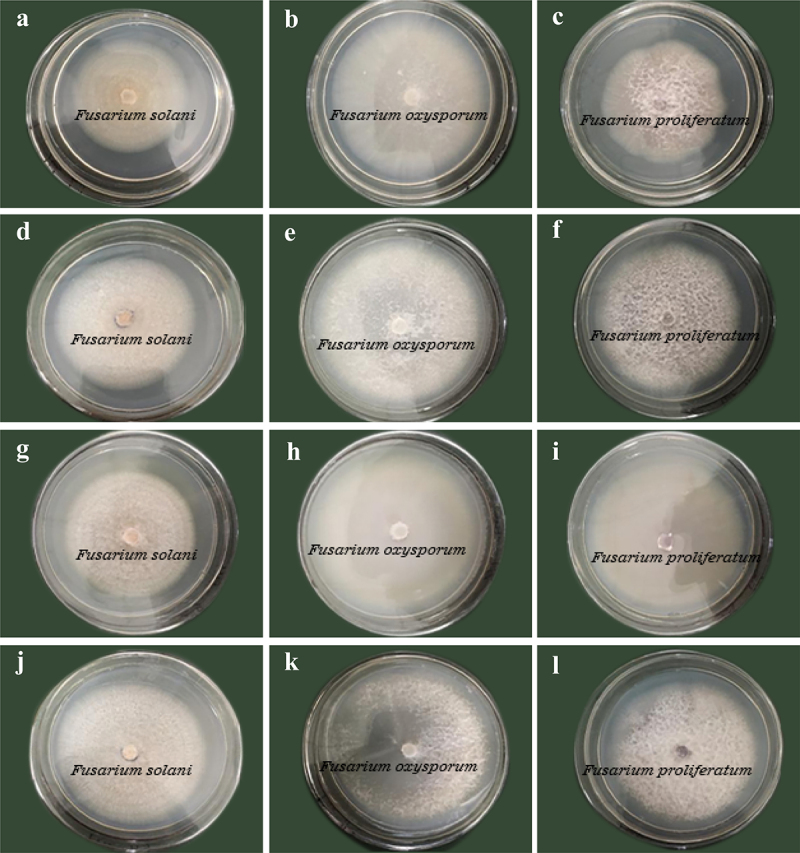
Antagonistic activities of the culture filtrates of antagonistic fungi against pathogens. a-c are T. asperellum against F. solani, F. oxysporum and F. proliferatum, respectively. d-f are T. hamatum against F. solani, F. oxysporum and F. proliferatum, respectively. g-i are T. virens against F. solani, F. oxysporum and F. proliferatum, respectively. j-l are the blank controls of F. solani, F. oxysporum and F. proliferatum, respectively.

**Figure 5.**
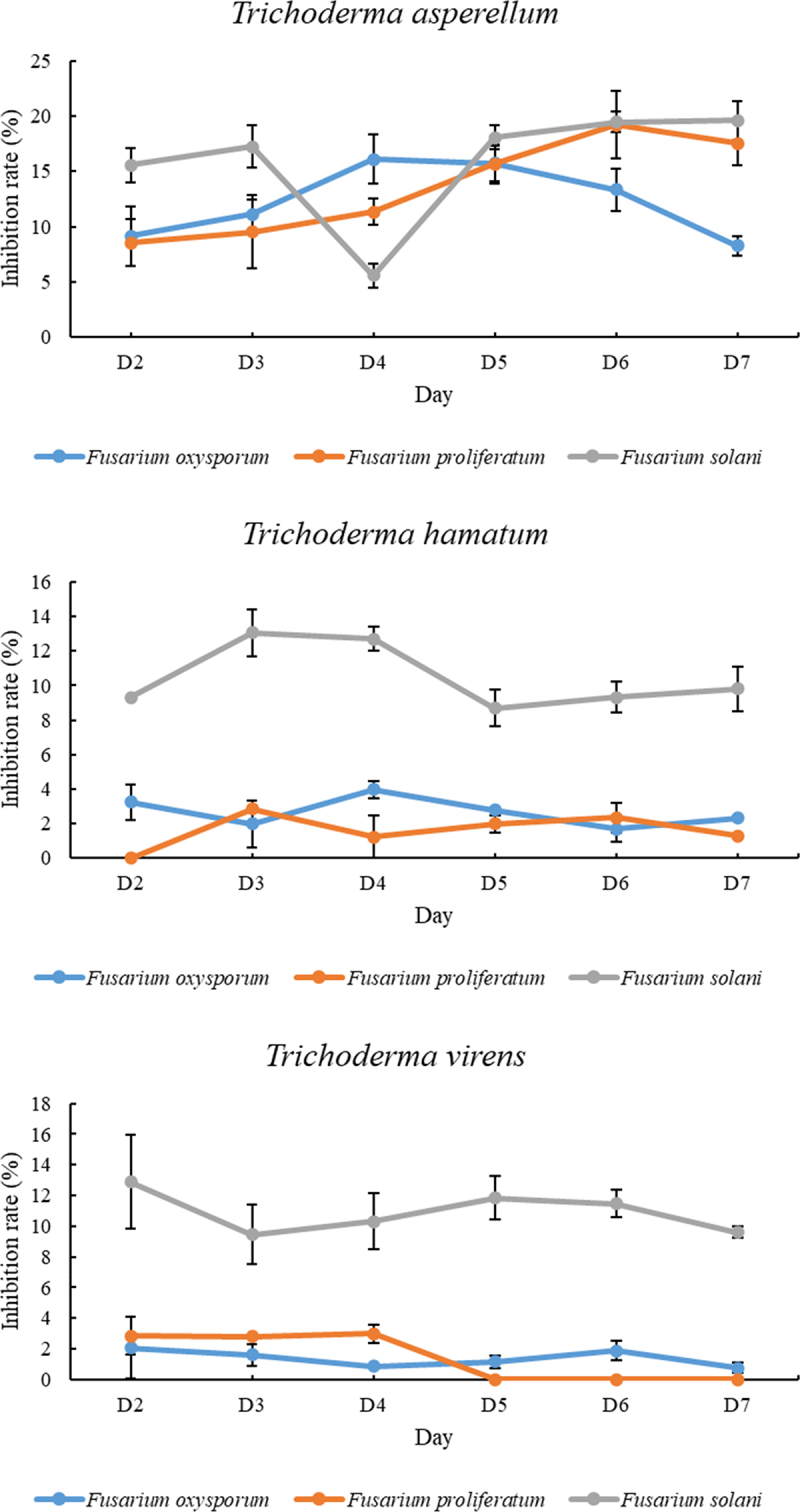
Inhibition rates of the culture filtrates of antagonistic fungi on the growth of pathogens.

As shown in Figure 4, on the 7th day of culture, the culture filtrates of the *Trichoderma* strains had weak inhibitory effects on the mycelial growth of the pathogens, and all the percentages of growth inhibition were below 20%. As shown in Figure 5, from day 2 to day 7, the antagonistic effect of *T. asperellum* on *F. oxysporum* increased quickly and peaked on day 4 at 16.10% and then decreased gradually. In contrast, the inhibitory effect of *T. asperellum* on *F. solani* showed a v-shaped trend and was the weakest on day 4 at only 5.55%, while the inhibitory effect was basically maintained at approximately 19.50% on day 6 and day 7. The inhibitory effect of *T. asperellum* on *F. proliferatum* was the highest (19.21%) on day 6. Compared with *T. asperellum*, *T. hamatum* and *T. virens* had weaker inhibitory effects on the pathogens, and both had better inhibitory activities against *F. solani*; however, the inhibition rates were only 8–14%. After day 4, the inhibitory activity of *T. virens* on *F. proliferatum* was no longer manifested. Although the colony diameter of the pathogen did not change significantly, the hyphae of the pathogen were degraded, indicating that *T. virens* had a certain antagonistic effect on *F. proliferatum*.

### 3.5. Impacts of nonvolatile metabolites on pathogen spore germination

The culture filtrates of *Trichoderma* were prepared to examine their effects on the germination of pathogenic spores. Table 3 shows that the three *Trichoderma* strains exhibited inhibitory effects on spore germination during the first 2 hours of cocultivation. In the subsequent observations, there was almost no inhibitory activity. According to the antagonistic activities of the culture filtrates of *Trichoderma* on mycelial growth and spore germination of pathogens, the nonvolatile metabolites had weak inhibitory activities on pathogens, indicating that the antagonistic components were mainly volatile metabolites.

**Table 3.** Inhibitory activities of the culture filtrates of antagonistic fungi on spore germination of pathogens.

Strains		Germination rates at different times (%)			
		2 h	4 h	6 h	8 h
*F. solani*	CK	9.90 ± 0.53a	17.67 ± 1.33c	67.18 ± 1.14a	71.35 ± 2.36a
	*T. asperellum*	7.55 ± 0.89b	37.29 ± 1.73b	44.33 ± 1.42c	71.75 ± 1.51a
	*T. hamatum*	5.23 ± 0.28c	43.78 ± 3.19a	58.85 ± 1.81b	71.53 ± 2.44a
	*T. virens*	3.41 ± 0.57d	46.54 ± 2.39a	63.92 ± 3.97a	71.52 ± 3.30a
*F. oxysporum*	CK	2.84 ± 0.42a	18.85 ± 1.71a	52.05 ± 6.57a	82.27 ± 5.34a
	*T. asperellum*	0.78 ± 0.23c	20.37 ± 2.11a	55.10 ± 5.35a	82.47 ± 3.18a
	*T. hamatum*	1.53 ± 0.30b	19.92 ± 5.82a	53.16 ± 4.73a	87.49 ± 2.45a
	*T. virens*	2.11 ± 0.38b	19.19 ± 4.26a	55.67 ± 0.31a	80.65 ± 2.94a
*F. proliferatum*	CK	4.23 ± 0.59a	14.86 ± 2.64a	54.06 ± 2.37a	91.47 ± 2.44a
	*T. asperellum*	3.19 ± 0.77a	18.15 ± 4.47a	55.11 ± 2.82a	93.29 ± 0.49a
	*T. hamatum*	3.81 ± 0.29a	17.82 ± 1.84a	50.76 ± 3.69a	94.77 ± 1.23a
	*T. virens*	3.63 ± 0.44a	17.46 ± 2.86a	55.07 ± 1.14a	93.54 ± 1.45a

Data in the table are mean ± standard deviation (n = 3). Different treatments marked with the same letters indicate no significant difference (P > 0.05), while different letters indicate significant or extremely significant differences (P < 0.05).

### 3.6. Analysis of volatile metabolites of antagonistic fungi by GC‒MS

**Figure 6.**
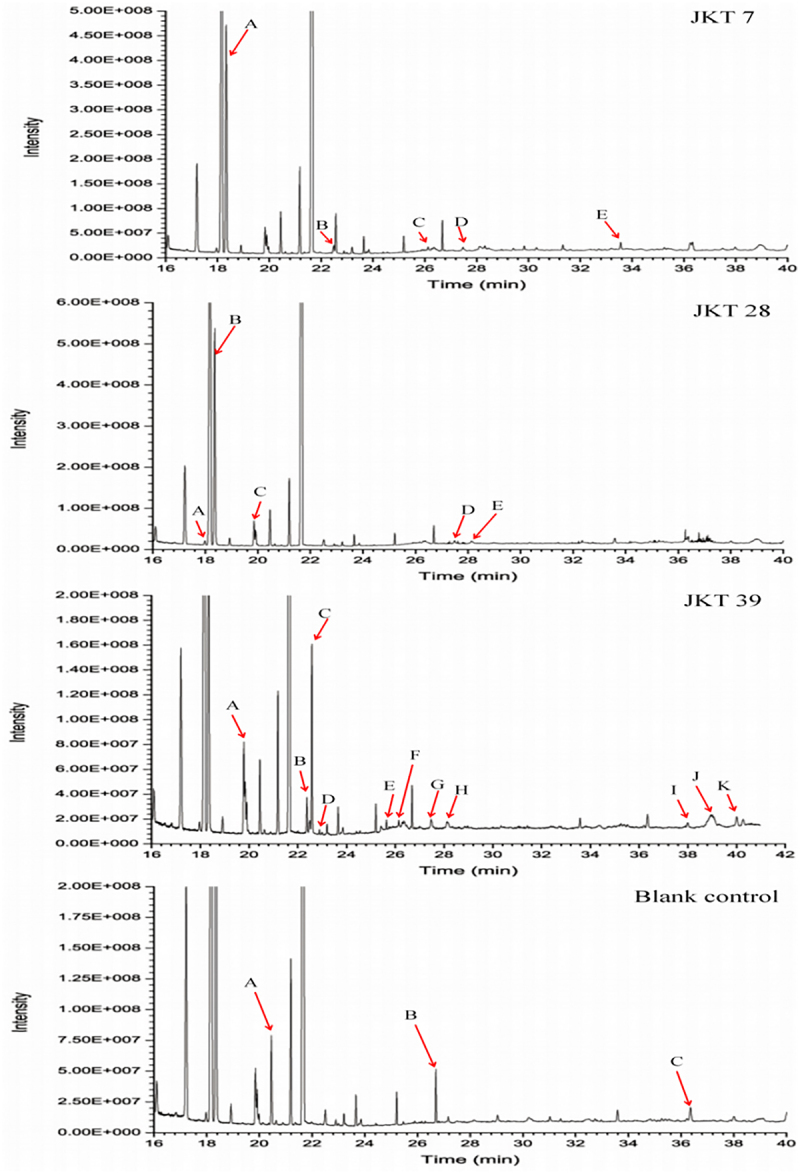
Chromatogram of volatile metabolites of ethyl acetate extract in the culture filtrates of antagonistic fungi.

**Figure 7.**
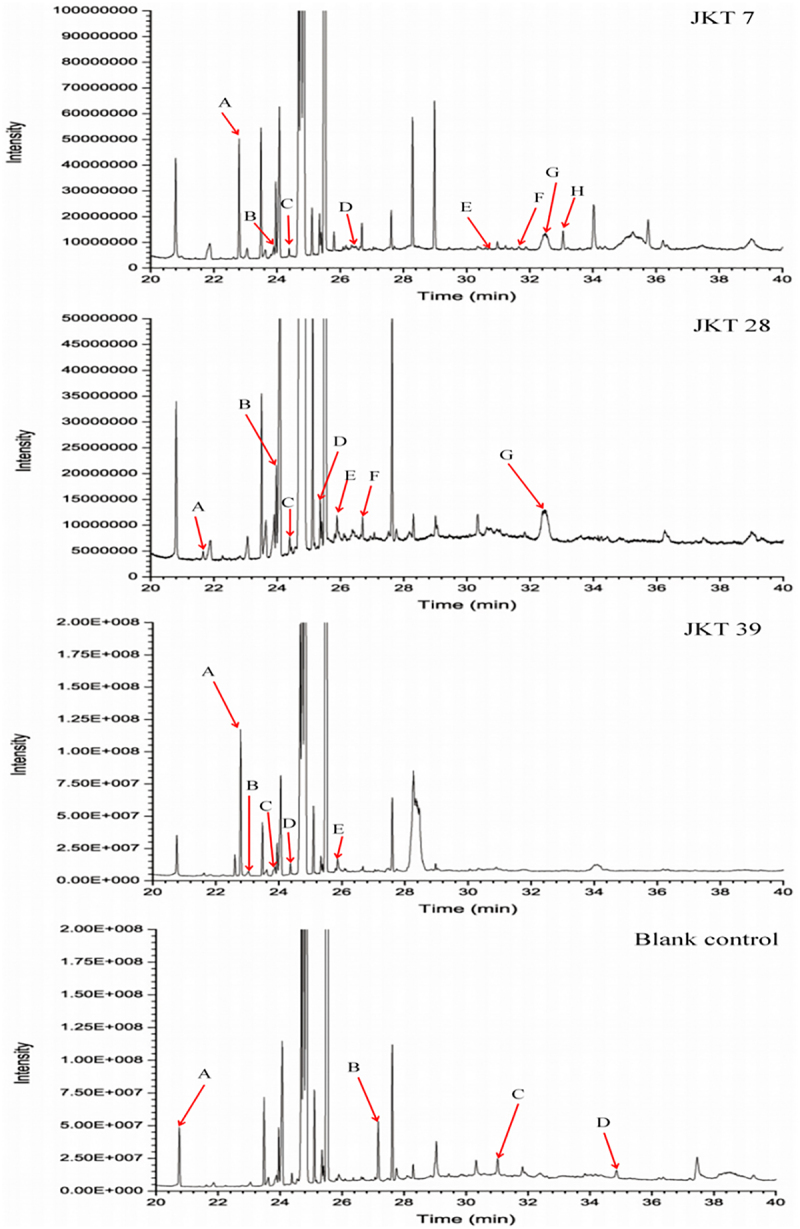
Chromatogram of volatile metabolites of n-butyl alcohol extract in the culture filtrates of antagonistic fungi.

The components of the culture filtrates extracted by ethyl acetate are shown in Figure 6 and Table 4. The results showed that the volatile metabolites of the ethyl acetate extract were mainly ketones, esters, alcohols and alkanes. The compositions of the culture filtrates extracted by n-butyl alcohol are shown in Figure 7 and Table 5. The volatile organic compounds were mainly silanes, ketones and alcohols.

**Table 4.** Volatile components of ethyl acetate extract in the culture filtrates of antagonistic fungi.

Strains	Peak No	Chemical name	Retention time (min)	Relative contents (%)
*T. asperellum*	A	Propyl acetate	18.334	8.98
	B	(2 R, 3 R)-2, 3-Butanediol	22.562	1.08
	C	4, 5-Octanediol	26.128	0.12
	D	Octadecane	27.479	0.29
	E	Pentadecane	33.557	0.31
*T. hamatum*	A	3-Methyl-2-butanone	17.986	0.21
	B	Propyl acetate	18.363	11.21
	C	Isopropyl acid ethyl ester	19.853	0.92
	D	Octadecane	27.493	0.20
	E	2, 6, 10, 14-Tetramethyl hexadecane	28.128	0.28
*T. virens*	A	3-Hydroxy-2-butanone	19.785	1.39
	B	2, 3-Butanediol	22.369	0.46
	C	L-(+)-2, 3-Butanediol	22.573	2.69
	D	n-Butyl ether	22.889	0.07
	E	3-Octanone	25.633	0.12
	F	4, 5-Dihydroxy octanone	26.139	0.08
	G	Octadecane	27.465	0.27
	H	2, 6, 10, 14-Tetramethyl hexadecane	28.142	0.25
	I	2, 6, 10-Trimethyl pentadecane	37.997	0.12
	J	Docosanoic	38.957	1.18
	K	Heptadecane	40.000	0.29
CK	A	Isobutyl Acetate	20.455	0.15
	B	Undecane	26.701	0.07
	C	Hexadecane	36.359	0.04

**Table 5.** Volatile components of n-butyl alcohol extract in the culture filtrates of antagonistic fungi.

Strains	Peak No	Chemical name	Retention time (min)	Relative contents (%)
*T. asperellum*	A	2, 3-Butanediol	22.803	0.79
	B	2, 4-Dimethyl-3-hexanone	23.892	0.06
	C	1, 3-(t-Butyldimethylsiloxy)-propylene glycol	24.379	0.05
	D	Dimethyl, isobutoxysilane	26.687	0.15
	E	Diethyl, ethoxy, isobutoxysilane	30.973	0.06
	F	Hexanolactam	31.872	0.03
	G	Dibutyl phthalate	32.492	0.69
	H	Dimethyl, pentyloxy, hexyloxy silane	33.055	0.19
*T. hamatum*	A	2-Propyne-1-alcohol	21.667	0.05
	B	2, 4-Dimethyl-3-hexanone	23.910	0.40
	C	1, 3-(t-Butyldimethylsiloxy)-propylene glycol	24.390	0.06
	D	3-Methyl-4-heptanol	25.358	0.30
	E	1, 3-Dihydroxyacetone	25.891	0.13
	F	Dimethyl, isobutoxysilane	26.698	0.09
	G	Dibutyl phthalate	32.449	0.90
*T. virens*	A	2, 3-Butanediol	22.788	1.87
	B	trans-1-butoxy-1-butene	23.039	0.13
	C	2, 4-Dimethyl-3-hexanone	23.878	0.20
	D	3-Methyl-4-heptanol	24.673	0.17
	E	1, 3-Dihydroxyacetone	25.870	0.23
CK	A	2-Methyl butanol	20.749	0.61
	B	Phenylacetaldehyde	27.171	0.51
	C	5-Hydroxymethylfurfural	31.016	0.18
	D	4-Propylphenol	34.854	0.13

## 4. Discussion

According to the field investigation of *A. carmichaelii* Debx. in the GAP production base (Jiangyou, China), local herbal farmers currently have no effective biological agents to control aconite root rot. The incidence of the disease can be reduced only by the timely removal of the diseased plants. To provide effective biocontrol for aconite root rot, three fungal strains, *T. asperellum*, *T. hamatum* and *T. virens*, showed significant antagonistic effects on pathogens of aconite root rot through a dual culture assay. After 3 days of dual culture, the inhibition rates of *T. asperellum* against *F. solani*, *F. oxysporum* and *F. proliferatum* were 37.51%, 43.18% and 37.14%, respectively. The inhibition rates of all three *Trichoderma* species against aconite root rot pathogens after 7 days of incubation increased to approximately 70%, which were slightly higher than those reported in the literature.^36–39^ The different antagonistic effects may be due to different strains of *Trichoderma* and pathogens.

The antagonistic effects of the volatile metabolites from the three *Trichoderma* fungi on the growth of pathogen mycelia were studied. The antagonistic effect was the highest on the 4th and 5th days of incubation, and the growth rates were reduced by the inhibitory effects in the following 48 hours. On the 7th day of culture, the inhibition rates of the three *Trichoderma* strains ranged from 12.73% to 62.09%. In the study of Zhang et al.,^40^ the antagonism rates of volatile metabolites of eleven *Trichoderma* strains against *F. oxysporum* ranged from 20.83% to 35.29%, among which the volatile substances produced by *T. harzianum* 223-2M1 had the best inhibitory effect on *F. oxysporum*. However, in this study, the inhibition rates of the three *Trichoderma* strains on *F. oxysporum* ranged from 12.73% to 37.00%. This may have been due to the different types and contents of volatile components produced by *Trichoderma* strains that grow in different environments, resulting in different antagonistic effects.

The culture filtrates of three *Trichoderma* strains showed weak antagonistic effects against the mycelial growth of pathogens, with all rates below 20%. The inhibitory activities of the culture filtrates on the spore germination of pathogens were mainly manifested in the first 2 hours of cocultivation, and no inhibition was observed after 2 hours. Therefore, it was speculated that the nonvolatile components in the culture filtrates were not the main effective components that inhibited the growth of the pathogens.

The volatile metabolites of antagonistic fungi were extracted and analyzed by GC‒MS. A total of thirteen volatile components were detected in the culture filtrates of *T. asperellum* and included four different alcohols as well as esters, silanes, alkanes, ketones and amines. The relative contents of esters and alcohols were higher than those of other components, with values of 9.67% and 2.04%, respectively. A total of twelve volatile components were detected in the culture filtrates of *T. hamatum*, which were ketones, esters, alcohols, alkanes and silanes. Among them, the relative content of esters was the highest, accounting for 13.03%. In the culture filtrates of *T. virens*, a total of fifteen volatile components were detected, which were six different ketones, followed by alkanes, alcohols, ethers and alkenes. The relative contents of alcohols and ketones were higher than those of other components, at 5.02% and 2.19%, respectively. The types and contents of volatile metabolites from different *Trichoderma* species were different. The compound octadecane analyzed by GC‒MS may have antifungal activities against *F. oxysporum* and *F. solani*, as well as the compound heptadecane against *F. oxysporum*.^41,42^ Hence, the antifungal activities of the three *Trichoderma* strains in this study may be related to the presence of these compounds.

The three *Trichoderma* strains screened in this research showed significant inhibitory effects on the growth of aconite root rot pathogens in the dual culture assay. Their volatile metabolites showed better inhibitory effects than the nonvolatile metabolites, which was different from the results of other scholars on the antagonistic effects of *Trichoderma*. For example, the antagonistic effects of *Trichoderma* T38D on soybean root rot pathogens were studied by Zhao et al.^43^ They found that the inhibitory effects of the metabolites produced by T38D that were difficult to volatilize were much stronger than those of the easily volatilized metabolites, which might be related to the different species of *Trichoderma* and the different hosts of pathogens.

## 5. Conclusions

The three *Trichoderma* strains screened in this study, *T. asperellum*, *T. hamatum* and *T. virens*, could inhibit the growth of aconite root rot pathogens. Their volatile metabolites showed better inhibition of pathogen growth than the nonvolatile metabolites.

These results highlight the potential advantage of these three *Trichoderma* strains as biocontrol agents against aconite root rot, and their secondary bioactive metabolites might be utilized as alternatives to synthetic fungicides to manage medicinal plant diseases.
